# Construction of Novel Nanocomposites (Cu-MOF/GOD@HA) for Chemodynamic Therapy

**DOI:** 10.3390/nano11071843

**Published:** 2021-07-16

**Authors:** Ya-Nan Hao, Cong-Cong Qu, Yang Shu, Jian-Hua Wang, Wei Chen

**Affiliations:** 1 Department of Chemistry, College of Sciences, Northeastern University, Shenyang 110819, China; neuhaoyanan@163.com (Y.-N.H.); qcc1531486377@163.com (C.-C.Q.); 2 Departments of Physics, University of Texas at Arlington, Arlington, TX 76019, USA; 3 Medical Technology Research Centre, Chelmsford Campus, Anglia Ruskin University, Chelmsford CM1 1SQ, UK

**Keywords:** Fenton reaction, hydroxyl radicals, GSH depletion, hydrogen peroxide, glucose oxidase

## Abstract

The emerging chemodynamic therapy (CDT) has received an extensive attention in recent years. However, the efficiency of CDT is influenced due to the limitation of H_2_O_2_ in tumor. In this study, we designed and synthesized a novel core-shell nanostructure, Cu-metal organic framework (Cu-MOF)/glucose oxidase (GOD)@hyaluronic acid (HA) (Cu-MOF/GOD@HA) for the purpose of improving CDT efficacy by increasing H_2_O_2_ concentration and cancer cell targeting. In this design, Cu-MOF act as a CDT agent and GOD carrier. Cu(II) in Cu-MOF are reduced to Cu(I) by GSH to obtain Cu(I)-MOF while GSH is depleted. The depletion of GSH reinforces the concentration of H_2_O_2_ in tumor to improve the efficiency of CDT. The resultant Cu(I)-MOF catalyze H_2_O_2_ to generate hydroxyl radicals (·OH) for CDT. GOD can catalyze glucose (Glu) to supply H_2_O_2_ for CDT enhancement. HA act as a targeting molecule to improve the targeting ability of Cu-MOF/GOD@HA to the tumor cells. In addition, after loading with GOD and coating with HA, the proportion of Cu(I) in Cu-MOF/GOD@HA is increased compared with the proportion of Cu(I) in Cu-MOF. This phenomenon may shorten the reactive time from Cu-MOF to Cu(I)-MOF. The CDT enhancement as a result of GOD and HA effects in Cu-MOF/GOD@HA was evidenced by in vitro cell and in vivo animal studies.

## 1. Introduction

Chemodynamic therapy (CDT) is an emerging cancer treatment, which depends on the Fenton or Fenton-like reactions to obtain highly toxic hydroxyl radicals (·OH) for killing cancer cells [1]. The Fe/hydrogen peroxide (H_2_O_2_) system is defined as the Fenton reagent, and the others (e.g., Co, Cd, Cu, Ag, Mn, Ni) are called Fenton-like reagents [2,3,4,5,6]. Compared with normal cells, the tumor microenvironment is characterized by overexpression of H_2_O_2_, glutathione (GSH) and weak acidity [7,8]. Up to now, most of the metal ions used to construct CDT nanotherapeutics include Mn, Fe and Cu. Fe ions in particular are the most commonly used for CDT [9]. However, the Fe-based Fenton reaction is only effective under strongly acidic conditions (pH 2–4) [10]. Therefore, the Fenton-reaction of Fe will be limited under the neutral and weakly acidic microenvironment conditions encountered in tumors. Mn(II) used in Fenton-like reactions only remains stable when pH < 4. In contrast, the efficiency of Cu(I) is not influenced by the pH. Even under the best reaction conditions, the reaction rate of Cu(I) is 160 times than that of Fe(II) [11,12,13]. In addition, a large amount of GSH in tumor cells can react with reactive oxygen species and affect the concentration of H_2_O_2_ and ·OH. GSH can be depleted during the reduction of the Cu(II) to Cu(I), which is beneficial for CDT [14]. Therefore, Cu-based nanotherapeutic agents for CDT have attracted a lot of attention.

The relatively higher concentration of H_2_O_2_ in tumor cells than in normal cells is the basis for CDT due to Fenton/Fenton-like reactions with H_2_O_2_. However, the H_2_O_2_ concentration in tumor cells is still limited and this limitation actually influences the efficiency of CDT [15]. Different kinds of strategies have been proposed to overcome this drawback. The introduction of extraneous enzyme (glucose oxidase (GOD), superoxide dismutase and so on) into the cell interior may facilitate the production of H_2_O_2_, which ensures the continuous and effective treatment of tumor cells [16]. GOD can catalyze the reaction of glucose (Glu) to produce gluconic acid and H_2_O_2_ [16,17]. Therefore, combining GOD and a CDT agent is a promising strategy. GOD consumes a large amount of Glu needed for physiological activities, which achieves a “cell starving” effect therapy during this process. This phenomenon also supplies H_2_O_2_ for CDT [18].

Metal-organic frameworks (MOFs) are organic-inorganic hybrid materials formed by the combination of inorganic metal ions or metal clusters and organic ligands. MOFs have been widely used in biomedical imaging and therapy [19], biosensing [20], catalysis and as drug carriers [21] due to their high specific surface area, porosity and diversified structures. MOFs can be easily modified with appropriate treatments during or after their synthesis [22]. Therefore, Cu-MOFs are suitable as modifiable CDT agents.

Hyaluronic acid (HA) is a natural acid mucopolysaccharide present in the synovial fluid and extracellular matrix [23]. HA has been widely used in tissue engineering, drug delivery and molecular imaging which all benefit from its biocompatibility and biodegradability [23,24,25]. More importantly, HA can specifically target CD44 overexpressed in various cancer cells and be decomposed by the intracellular hyaluronidase [26]. Therefore, HA is often used to bounding various drug-loaded nanoparticles as a targeting moiety for enhanced cancer therapy [27,28,29,30].

**Figure 1:**
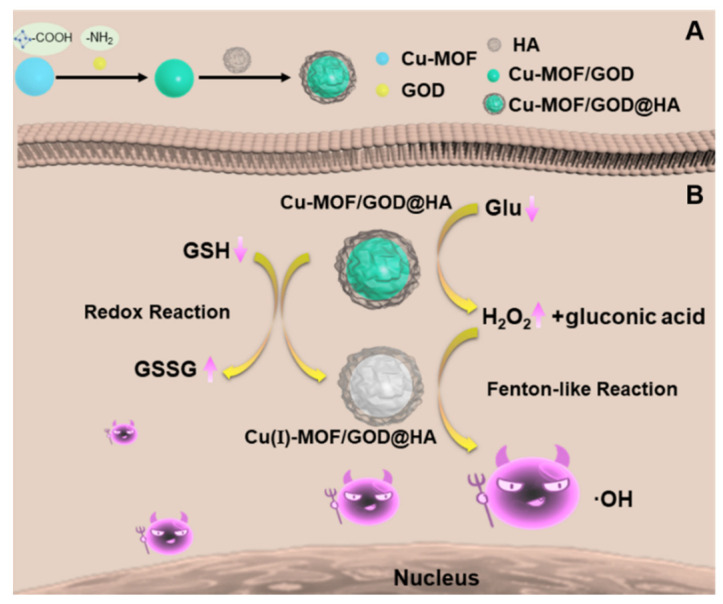
Schematic of the Cu-MOF/GOD@HA preparation process (**A**) and the Cu-containing nanoformulation mediated CDT (**B**).

In this work, Cu-MOF are chosen as a cascade nanoreactor for CDT. As shown in Figure 1, GOD are first loaded onto Cu-MOF and Cu-MOF/GOD composites are thus obtained. HA acts as a shell on the Cu-MOF/GOD to avoid GOD leakage and as a targeting molecule to tumor site. The resulting Cu-MOF/GOD@HA nanocomposites are activated by GSH in tumors and catalyze H_2_O_2_ to produce ·OH for CDT for cancer treatment as illustrated in Figure 1.

## 2. Materials and Methods

### 2.1. The Preparation of Cu-MOF/GOD@HA Nanocomposites

#### 2.1.1. Cu-MOF/GOD

Cu-MOFs (HKUST-1) were prepared according to a previously reported approach [31]. Thus, 10 mg of Cu-MOF were dissolved in 1 mL of absolute ethanol. Next 10 mg of coupling agent (1-(3-dimethylaminopropyl)-3-ethylcarbodiimide hydrochloride/N-hydroxysuccinimide) (EDC/NHS) were dissolved in 6 mL of DI water. The above Cu-MOF and EDC/NHS solutions were mixed with vigorous stirring for 30 min. Then 2 mg of GOD was dissolved in 3 mL of DI water and added dropwise into the above mixture under stirring for 4 h. Finally, the resultant Cu-MOF/GOD was washed with DI water three times.

#### 2.1.2. Cu-MOF/GOD@HA

The above Cu-MOF/GOD nanoparticles (final concentration: 2 mg mL^–1^) mixed with HA (final concentration: 0.5 mg mL^–1^) under sonication for 30 min. Finally, the resultant Cu-MOF/GOD@HA was washed with DI water 3 times.

### 2.2. Extracellular ·OH Generation under Catalysis by Cu-MOF

#### 2.2.1. Reaction between Cu-MOF and GSH

Two hundred μL of Cu-MOF solution (1 mg mL^–1^) was mixed with 800 μL GSH solution (10 mmol L^–1^) under vortex mixing until the appearance of a white precipitate (Cu(I)-MOF). The precipitated intermediate products were characterized via UV-vis absorption spectroscopy (Hitachi, Hitachi, Japan).

#### 2.2.2. Extracellular ·OH Generation under Catalysis by Cu-MOF

A 5 μg mL^–1^ methylene blue (MB) solution was mixed with 5 mmol L^–1^ H_2_O_2_ and 200 μg mL^–1^ Cu(I)-MOF or Cu-MOF under vortex mixing. After different time intervals of reaction, ·OH-induced MB degradation was evaluated based on the change in the absorption at the maximum absorption wavelength 630 nm.

#### 2.2.3. Gluconic Acid Generation under Catalysis by GOD

(1) A 100 μg mL^–1^ Cu-MOF or Cu-MOF/GOD solution was mixed with 500 μg mL^–1^ Glu under vortex mixing. After different times of reaction, the generation of gluconic acid was monitored by the acidity change of the solution. (2) A 100 μg mL^–1^ Cu-MOF or Cu-MOF/GOD solution was mixed with different concentrations of Glu under vortex mixing for 24 h. The generation of gluconic acid was monitored by the acidity change of the solution.

#### 2.2.4. The H_2_O_2_ Generation under Catalysis by GOD

The fluorescence signals of Ampliflu Red are influenced by the change of H_2_O_2_ concentration. Thus, Ampliflu Red was used to investigate the H_2_O_2_ generation. Specific steps were as follows: (1) A 600 μg mL^–1^ Ampliflu Red solution was mixed with 500 μg mL^–1^ Glu and 100 μg mL^–1^ Cu-MOF/GOD under vortex mixing. After different time intervals (0, 5, 10, 25, 50 min), the fluorescence intensity at ∽585 nm under 530 nm wavelength excitation was measured. (2) A 600 μg mL^–1^ Ampliflu Red solution was mixed with 100 μg mL^–1^ Cu-MOF/GOD and different concentrations of Glu (0, 20, 50, 70, 100 μg mL^–1^) under vortex mixing. After 30 min of reaction, the fluorescence intensity at ∼585 nm under 530 nm wavelength excitation was measured.

### 2.3. Cell Experiments

#### 2.3.1. Cytotoxicity Assay

Briefly, 10^4^ cells/well were seeded in 96-well plates and cultured overnight at 37 °C in 5% CO_2_ atmosphere. The pending test cells were then further incubated with different concentrations of Cu-MOF, Cu-MOF/GOD or Cu-MOF/GOD@HA (10, 20, 30, 50, 70, 100 μg mL^−1^) under different conditions (GSH: 2.5, 5.0 mmol L^–1^, H_2_O_2_: 100 µmol L^–1^ or trypan blue: 10 μg mL^–1^) for 24 h. The cytotoxicity of Cu-MOF, Cu-MOF/GOD and Cu-MOF/GOD@HA was evaluated through an MTT assay.

#### 2.3.2. Intracellular ·OH Generation Capability of Cu-MOF/GOD@HA

For the evaluation of ·OH generation capability, MCF-7 cells were cultured with 50 μg mL^–1^ of Cu-MOF, Cu-MOF/GOD and Cu-MOF/GOD@HA for 4 h, the cellular-ROS stress levels were then evaluated by using a ROS assay kit. After washing with PBS for twice, MCF-7 cells were further incubated with 2′,7′-dichlorodihydrofluorescein diacetate (DCFH-DA) (10 μmol L^–1^) for 20 min followed by washing for 3 times with PBS. Finally, ROS associated signals in the cells were observed by fluorescence microscopy.

## 3. Results

### 3.1. Preparation and Characterization of Cu-MOF/GOD@HA

The preparation process of Cu-MOF/GOD@HA was shown in Figure 1A as described in the experimental section. GOD was firstly loaded into Cu-MOF by amide reaction between –COOH on the Cu-MOF and –NH_2_ on GOD. Then, Cu-MOF/GOD was coated by HA to avoid the leakage of GOD and to improve its biocompatibility as well as the targeting ability. The CDT mechanism of Cu-MOF/GOD@HA was shown in Figure 1B. After endocytosis into MCF-7 cells, i.e., the tumor cells, CDT process based on Cu-MOF/GOD@HA was triggered sequentially by GSH “AND” H_2_O_2_ in the cancer cell interior. In addition, GOD in Cu-MOF catalysis Glu to supply H_2_O_2_ to improve CDT efficacy.

**Figure 2:**
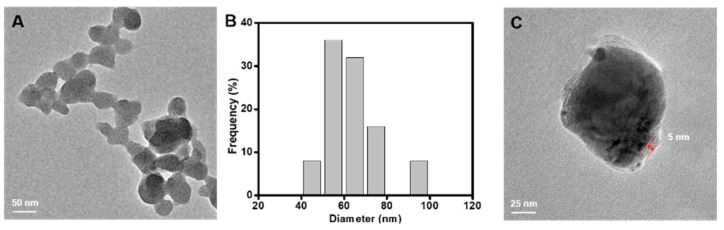
(**A**) TEM images of Cu-MOF. Scale bar: 50 nm. (**B**) The size distribution of Cu-MOF as measured by TEM image. (**C**) TEM images of Cu-MOF/GOD@HA (Scale bar: 25 nm).

Transmission electron microscope (TEM) images in Figure 2A,B illustrate the Cu-MOF with diameter of 63.32 ± 9.12 nm. SEM image and Elemental mapping of C, N, O and Cu of Cu-MOF confirmed the successful preparation (Figure S1A,B). From the EDS spectra from SEM, Cu-MOF were consisted of 1% Cu, 2% N, 5% O and 92% C. As shown in Figure 2C, the shells of loaded GOD and coated HA were 5 nm. Compared with Cu-MOF, the FT-IR spectra of Cu-MOF/GOD, Cu-MOF/GOD@HA and GOD showed bands at 1550 cm^–1^, attributed to vibrational stretches characteristic of GOD, indicating the successful loading of GOD (Figure S2A). Furthermore, the TGA analysis of Cu-MOF and Cu-MOF/GOD@HA further confirmed the GOD loading and HA coating (Figure S2B). From the TGA analysis, Cu-MOF/GOD@HA were consisted with 15.32% HA, 21.56% GOD and 63.12% Cu-MOF.

**Figure 3:**
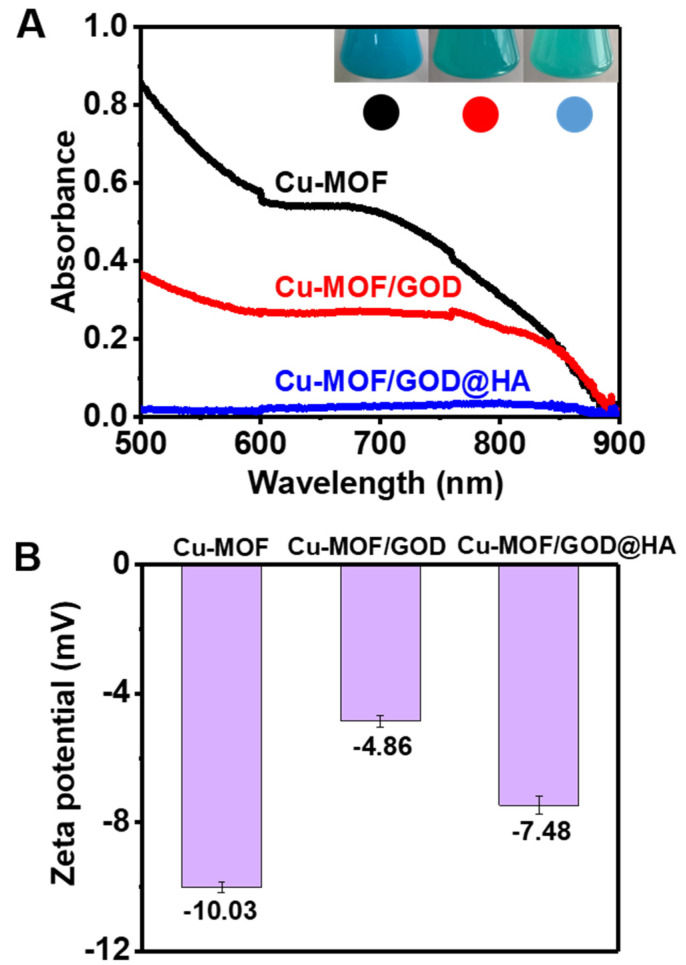
(**A**) UV-vis absorption spectra of Cu-MOF, Cu-MOF/GOD and Cu-MOF/GOD@HA (The concentrations of pending test samples were 100 μg mL^–1^). Inset in (**A**) shows the photographs of the solutions containing Cu-MOF, Cu-MOF/GOD and Cu-MOF/GOD@HA. (**B**) Zeta potential values of Cu-MOF, Cu-MOF/GOD and Cu-MOF/GOD@HA. (The concentrations of pending test samples are 200 μg mL^–1^).

In order to confirm the successful preparation of Cu-MOF/GOD@HA, the UV-vis absorption spectra of the reaction mixture were recorded. The absorption-change of Cu-MOF, Cu-MOF/GOD and Cu-MOF/GOD@HA in the 600–900 nm region, indicating the successful assembly of Cu-MOF/GOD@HA is clearly seen in Figure 3A. The color of Cu-MOF and Cu-MOF/GOD@HA changed from sky blue to green, also indicating successful loading of GOD and coating of HA. Zeta potential values of the corresponding nanocomposites obtained in each step are shown in Figure 3B, where the Cu-MOF aqueous solution exhibits a potential of −10.03 ± 0.16 mV. The loading of GOD reduces the zeta potential to −4.84 ± 0.19 mV, implying the deprotonation of the –NH_2_ of the GOD. The increase of zeta potential to −7.84 ± 0.28 mV due to the presence of the –OH of HA [32,33]. Furthermore, the X-ray diffraction (XRD) spectra of Cu-MOF, Cu-MOF/GOD and Cu-MOF/GOD@HA indicates that the loading of GOD and coating of HA did not influence the crystal structure of Cu-MOF (Figure S3).

### 3.2. Depletion of GSH and Generation of H_2_O_2_

In order to confirm the oxidation-reduction reactions between Cu-MOF and GSH, molar equivalents of Cu-MOF and GSH were mixed in an aqueous solution. It is clearly illustrated in Figure S4 that the absorption of Cu-MOF completely disappeared after reaction of Cu-MOF and GSH for ca. 2 min along with the formation of Cu(I)-MOF, and the color of the mixture of Cu-MOF and GSH turned from sky blue to white. This observation obviously demonstrated that the incubation of Cu-MOF with GSH facilitates the reduction of divalent Cu(II) in the Cu-MOF by GSH. In addition, compared with TEM image of Cu-MOF, Cu-MOF was collapsed after treatment with 10 mmol L^–1^ GSH (Figure S5), proving the reaction between GSH and Cu-MOF.

X-ray photoelectron spectroscopy (XPS) was also used to investigate the oxidation-reduction reactions between Cu-MOF and GSH. XPS is a surface sensitive technique. Compared with Cu-MOF (Figure S6A), an obvious N1s peak was observed in the XPS spectra of Cu-MOF/GOD and Cu-MOF/GOD@HA (Figure S6B,C), further demonstrating the occurrence of GOD loading. After treatment by GSH, an obvious N1s peak appears in Cu-MOF due to the residues of GSH (Figure S6D). Furthermore, after treatment of GSH, the XPS analysis of Cu(I)-MOF only shows Cu(I) (932.7, 952.4 eV) peaks (Figure S6E), also confirming the reduction of Cu-MOF by GSH. Moreover, Figure 3A also shows that after loading with GOD and coating with HA, the UV-vis absorption of between 600–900 nm is significantly reduced. Interestingly, the proportion of Cu(I) on the surface of Cu-MOF have increased after loading with GOD and coating with HA. As shown in Figure S6F–H, the valence state of Cu is “+1” or “+2”, since the Cu 2p XPS spectra displayed peaks on Cu(II) (934.4 eV, 954.2 eV)/Cu(I) (932.7 eV, 952.4 eV). The existence of Cu(II)/Cu(I) redox pair provide a great potential for Fenton-like reactions. In the paramagnetic chemical state (Figure S6F–H), the Cu 2p_3/2_ XPS spectrum is quantitatively analyzed, and the Cu(II)/Cu(I) ratio of Cu-MOF is 1.2872. The Cu(II)/Cu(I) ratios on the surface of Cu-MOF/GOD and Cu-MOF/GOD@HA in Cu 2p_3/2_ XPS spectra are 1.0376 and 0.8744, respectively. Obviously, after loading with GOD and coating with HA, the proportion of Cu(I) on the surface of Cu-MOF increased, indicating that Cu(II) was reduced to Cu(I) during the assembly process on the surface of Cu-MOF. This phenomenon may shorten the reaction time from Cu-MOF to Cu(I)-MOF.

**Figure 4:**
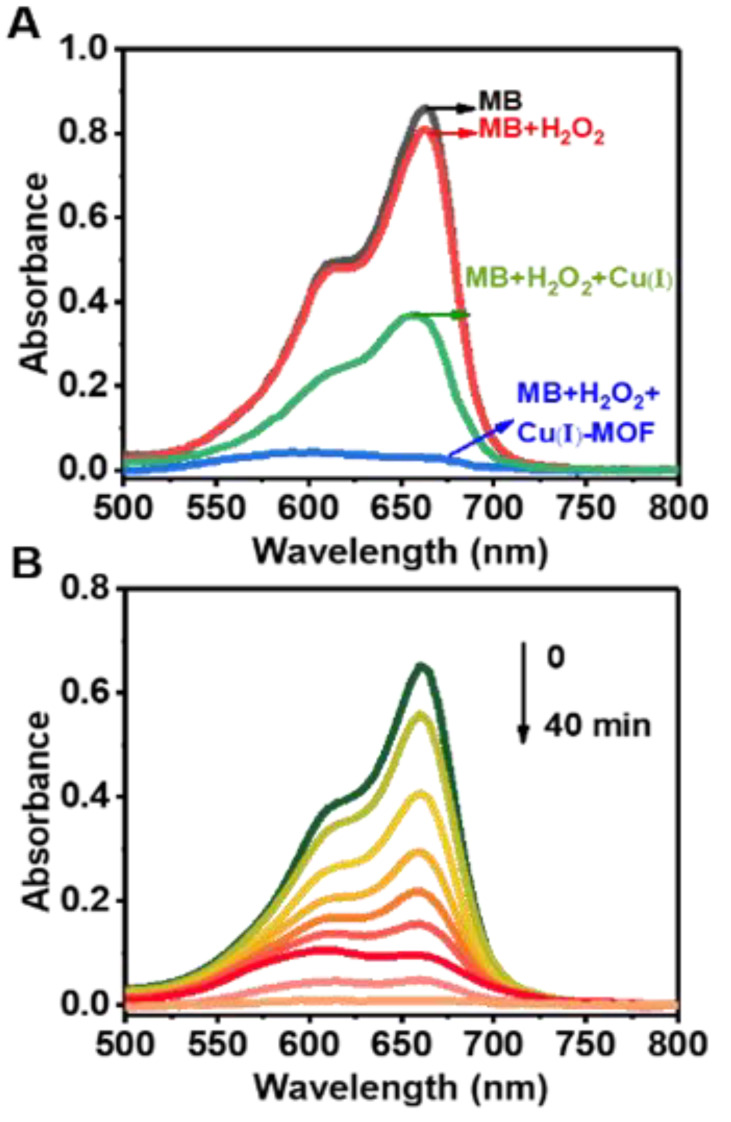
Experimental demonstration of the mechanism for the CDT process with Cu-MOF as the nano-therapeutic agent. OH generated from the Fenton-like reaction between Cu(I)-MOF and H_2_O_2_ leads to the degradation of MB. (**A**) MB degradation after incubation for 40 min in the presence of Cu(I)-MOF, H_2_O_2_, H_2_O_2_+Cu(I)-MOF and H_2_O_2_+free Cu(I), respectively. (**B**) The time-dependent degradation process of MB in the presence of Cu(I)-MOF. MB: 5 μg mL^–1^, H_2_O_2_: 10 mmol L^–1^, Cu(I)-MOF: 200 μg mL^–1^ (Cu(I): 0.494 μg mL^–1^), free Cu(I): 0.494 μg mL^–1^, time interval: 5 min.

After reduction of Cu-MOF by GSH, Cu(I) moiety catalyzes H_2_O_2_ to generate ·OH via a Fenton-like reaction. The Cu(I)-MOF-involved CDT processes are investigated and demonstrated by observing the degradation methylene blue (MB) [34]. ·OH induced degradation of MB which may result in a significant decrement on the absorption of MB at the maximum wavelength of 663 nm. Figure 4A shows that the degradation results of MB under different conditions by incubation for 40 min. In the presence of only Cu(I)-MOF or H_2_O_2_, virtually no degradation of MB was observed. On the contrary, a remarkable degradation of MB was recorded by introducing H_2_O_2_ into the mixture containing MB and Cu(I)-MOF. In addition, in the presence of free Cu(I) and H_2_O_2_, no remarkable degradation of MB was observed within 40 min. Thus, compared with free Cu(I), Cu(I) activated from Cu-MOF has a higher catalytic performance. Moreover, Figure S7 shows that Cu-MOF could not react with H_2_O_2_ to generate ·OH to reduce the degradation of MB. This provides a clear evidence for the occurrence of the Cu-MOF involving in the cascade reactions in CDT process. First, divalent Cu(II) in Cu-MOF was activated by GSH to convert to monovalent Cu(I) in Cu(I)-MOF. Subsequently, Cu(I)-MOF catalyzes the generation of ·OH from H_2_O_2_. The absorption spectra of MB illustrating its time-dependent degradation is shown in Figure 4B. It is obvious that MB was completely degraded after incubation for 40 min.

The depletion of GSH by Cu-MOF and the generation of H_2_O_2_ by GOD were investigated. First of all, Cu-MOF were treated by excess GSH. The residual GSH was indicated by 5,5′-dithiobis (2-nitrobenzoic acid) (DTNB), a kind of sulphydryl (–SH) indicator at different time points [35]. With the extended time, the characteristic absorbance at ~412 nm decreased, indicating the depletion of GSH. As shown in Figure S8A, the depletion capacity of GSH exhibits a concentration-dependent model. The fluorescence signals of Ampliflu Red were influenced by the change of H_2_O_2_ concentration [36]. Therefore, Ampliflu Red can be used to investigate the generation of H_2_O_2_. As shown in Figure S8B,C, the generation of H_2_O_2_ exhibits a time-dependent model and a Glu-concentration-dependent model. The catalytic reaction of GOD with glucose to produce gluconic acid and H_2_O_2_ will cause the pH of the aqueous solution to change. Therefore, after mixing Cu-MOF or Cu-MOF/GOD and Glu for different time, the pH change can reflect the catalytic performance of GOD in Cu-MOF/GOD. As shown in Figure S9, the pH values show a time-dependent mode and a concentration-dependent mode (Glu). These results indicate that GOD loaded in Cu-MOF/GOD still can catalyze Glu to produce H_2_O_2_.

### 3.3. Cell Experiment

After Cu-MOF/GOD@HA endocytosis into tumor cells, Cu(Ⅱ) in Cu-MOF were reduced to Cu(Ⅰ) by GSH in tumor. Cu(Ⅰ) catalyzes H_2_O_2_ to generate a large number of ·OH. The excessive ·OH oxidize signal molecules, cytokines, proteins, nucleic acids, carbohydrates, lipids and etc to promote apoptosis of tumor cells [1,2,4,5,7].

**Figure 5:**
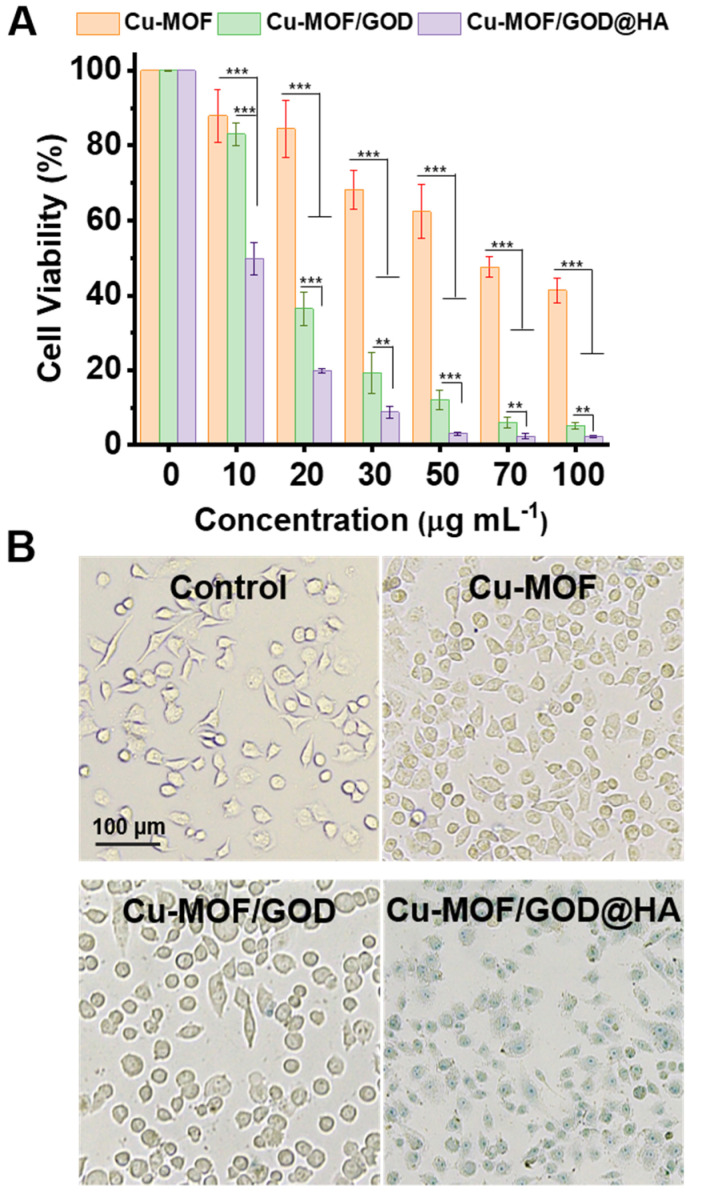
Cytotoxicity of different nanocomposites under different conditions. (**A**) MCF-7 cells were incubated with Cu-MOF, Cu-MOF/GOD and Cu-MOF/GOD@HA at concentrations of 0, 10, 20, 30, 50, 70, 100 µg mL^–1^. (**B**) Microscopy images of MCF-7 cells with trypan blue staining. MCF-7 cells were incubated with Cu-MOF, Cu-MOF/GOD and Cu-MOF/GOD@HA for 4 h at the levels of 20 µg mL^–1^. Scale bar: 100 µm. Values of *p* < 0.05 were considered statistically significant, with *, **, *** represent *p* < 0.05, *p* < 0.01 and *p* < 0.001, respectively.

MTT experiments were used to evaluate the lethality of Cu-MOF, Cu-MOF/GOD and Cu-MOF/GOD@HA [37,38,39,40]. The viabilities of MCF-7 cells treated with Cu-MOF, Cu-MOF/GOD and Cu-MOF/GOD@HA at several concentrations (0, 10, 20, 30, 50, 70, 100 µg mL^–1^) levels were calculated, as illustrated in Figure 4A. The quantitative results showed that 10 µg mL^–1^ the Cu-MOF/GOD@HA nanocomposites gave rise to an MCF-7 cell viability of 49.8%, while the same concentration of Cu-MOF and Cu-MOF/GOD produced an MCF-7 cell viability of 87.9% and 83.1%, respectively. This could be attributed to the targeting ability of HA. 20 µg mL^–1^ the Cu-MOF nanocomposites gave rise to an MCF-7 cell viability of 84.5%, while the same concentration of Cu-MOF/GOD and Cu-MOF/GOD@HA produced an MCF-7 cell viability of 36.5% and 19.9%, respectively. This could be due to the loading of GOD. GOD catalyzes Glu to supply more H_2_O_2_ for CDT. The 50% inhibitory concentration (IC_50_) values of Cu-MOF, Cu-MOF/GOD and Cu-MOF/GOD@HA for MCF-7 cells were found to be 70.8, 17.5, 9.7 µg mL^–1^. In general, Cu-MOF/GOD@HA improves its own tumor lethality in two aspects, one is the catalytic ability of GOD, and the other is the targeting ability of HA. When MCF-7 cells were incubated with lower concentration Cu-MOF/GOD@HA, the targeting performance of HA plays a dominant role. When MCF-7 cells were incubated with higher concentration Cu-MOF/GOD@HA, the catalysis performance of GOD plays a dominant role. Moreover, trypan blue was used to observe the lethality of Cu-MOF/GOD@HA more intuitively as illustrated in Figure 5B. It is clearly shown that the incubation of MCF-7 cells with Cu-MOF, Cu-MOF/GOD and Cu-MOF/GOD@HA for 4 h leads to the significant decrease on the number of cells and the morphology of MCF-7 cells became irregular. Catalytic ability of GOD and targeting ability of HA improve the lethality of Cu-MOF/GOD@HA.

### 3.4. In Vivo Antitumor Efficacy

**Figure 6:**
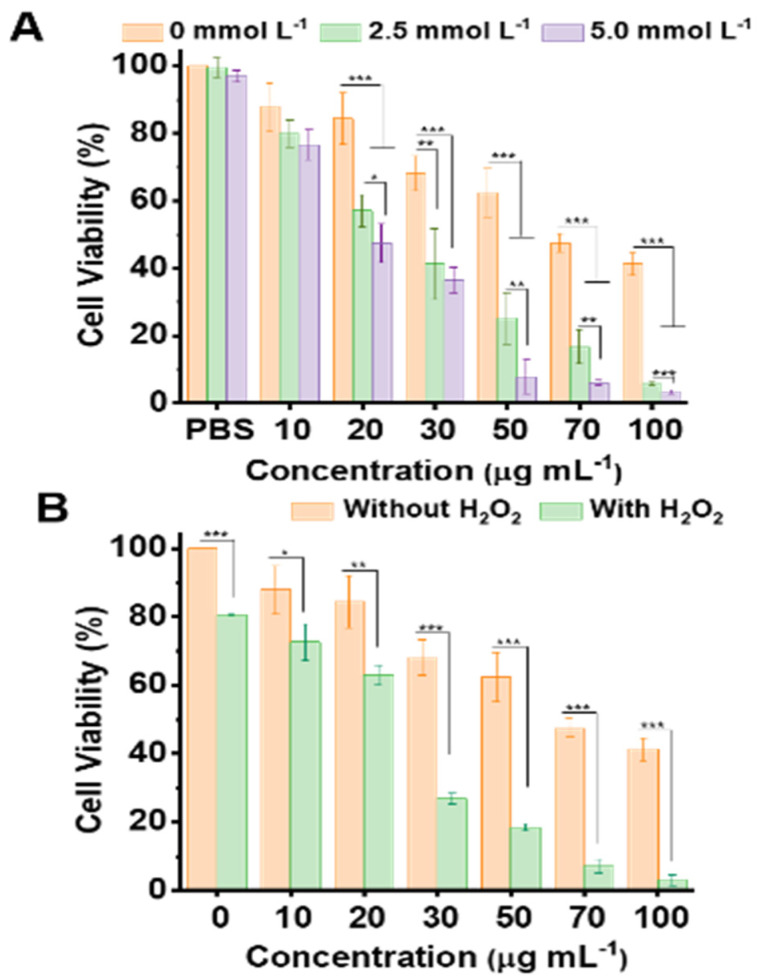
(**A**) The dependence of MCF-7 cell viability on the concentration of GSH (0, 2.5 and 5.0 mmol L^–1^) by incubation with Cu-MOF at the levels of 10, 20, 30, 50, 70, 100 µg mL^–1^. (**B**) The MCF-7 cell viability on H_2_O_2_ (100 µmol L^–1^) by incubation with Cu-MOF at the levels of 10, 20, 30, 50, 70, 100 µg mL^–1^. Values of *p* < 0.05 were considered statistically significant, with *, **, *** represent *p* < 0.05, *p* < 0.01, and *p* < 0.001, respectively.

The above discussions about the Cu-MOF/GOD@HA mediated CDT mechanisms indicate that the CDT process is significantly sensitive to GSH, which to a large extent determines the efficiency of CDT. In the present case, the variation of cell viability is evaluated by regulating the level of GSH in the cancer cell interior. For this purpose, MCF-7 cells were pre-incubated with GSH at the concentration of 2.5 and 5.0 mmol L^–1^, respectively. Then, the cytotoxicity of Cu-MOF was assessed. As shown in Figure 6A, the increase of GSH level in tumor cell environment leads to a significant increase on the lethality of Cu-MOF to MCF-7 cells. The corresponding IC_50_ values of Cu-MOF were 70.8, 24.0 and 19.1 µg mL^–1^, at GSH levels of 0, 2.5 and 5.0 mmol L^–1^, respectively. Moreover, the variation of cell viability was evaluated by regulating the level of H_2_O_2_ in the cancer cell interior. For this purpose, MCF-7 cells were pre-incubated with H_2_O_2_ at the concentration of 100 µmol L^–1^. Then, the cytotoxicity of Cu-MOF was assessed. As shown in Figure 6B, the increase of H_2_O_2_ level in the tumor cell environment leads to a significant increase in the lethality of Cu-MOF to MCF-7 cells. The corresponding IC_50_ values were 70.8 and 20.3 µg mL^–1^, at H_2_O_2_ levels of 0 and 100 µmol L^–1^. Furthermore, in order to investigate the influence of Cu-MOF, Cu-MOF/GOD and Cu-MOF/GOD@HA on the production of intracellular ROS, ROS detection kit was used for cell staining after MCF-7 cells were incubated with 50 µg mL^–1^ of Cu-MOF, Cu-MOF/GOD and Cu-MOF/GOD@HA for 4 h. The results in Figure S10 indicate that the loading of GOD and targeting ability of HA are beneficial for the generation of ·OH.

The in vivo antitumor efficacy of the Cu-MOF, Cu-MOF/GOD and Cu-MOF/GOD@HA was investigated in nude mice bearing MCF-7. Once the tumors had grown to approximately 90 mm^3^, the mice were intertumorally injected with either PBS (control, 10 mmol L^–1^), Cu-MOF, Cu-MOF/GOD and Cu-MOF/GOD@HA (The concentrations were all 2.5 mg kg^–1^) in 10 mmol L^–1^ PBS every 2 days (*n* = 3). A total of seven injections were performed over 2 weeks. During the treatment process, the mice in control (PBS) and experimental groups exhibit virtually no difference in their body weight, indicating negligible systemic toxicity of Cu-MOF, Cu-MOF/GOD and Cu-MOF/GOD@HA itself (Figure S11A). As shown in Figure S11B, tumor sizes in the PBS group were evidently increased from ~100 mm^3^ to ~774 mm^3^. the tumor sizes in the groups by injecting 2.5 mg kg^–1^ Cu-MOF, Cu-MOF/GOD were increased from ~90 mm^3^ to ~238, 95 mm^3^, respectively. On the contrary, the tumor sizes in the groups by injecting 2.5 mg kg^–1^ Cu-MOF/GOD@HA were decreased from ~90 mm^3^ to 45 mm^3^. At the end of the treatment (15 days), the tumor tissues were excised from the mice and weighed. As shown in Figure S11C, the tumor sizes in the experimental and groups (PBS, Cu-MOF, Cu-MOF/GOD and Cu-MOF/GOD@HA) decreased by 7.72, 2.98, 1.08 and 0.57 with respect to the initial-tumor volumes. The tumor sizes were obviously increased in the PBS group. In contract, the tumor sizes for the experimental group were remarkably decreased (Figure S11D).

In the concentration range of 10–100 µg mL^–1^, Cu-MOF/GOD@HA exhibited no obvious hemolytic effect, and the hemolytic rate at each concentration level was less than 4%. This result well indicated the excellent blood compatibility of the Cu-MOF/GOD@HA. Figure S11E illustrated that after H&E staining of the major organs of mice including heart, liver, spleen, lung and kidney, no obvious pathological changes in these tissues were observed in the presence of Cu-MOF/GOD@HA. In order to avoid the possible hemolysis or blood cell aggregation after intravenous injection, the hemolysis experiment of Cu-MOF/GOD@HA was carried out (Figure S12).

## 4. Conclusions

In summary, Cu-metal organic framework (Cu-MOF)/glucose oxidase (GOD)@hyaluronic acid (HA) (Cu-MOF/GOD@HA) were prepared for chemodynamic therapy (CDT) with the aims to increase H_2_O_2_, targeting and efficacy. Cu-MOF/GOD@HA were activated by intracellular GSH and catalyzed intracellular H_2_O_2_ reactions to generate ·OH. During these processes, a large amount of ·OH were generated which well facilitates CDT for cancer destruction. The loading of GOD and coating of HA can improve the efficacy of CDT. The present study provides a useful route for the development of nanotherapeutic agents for the treatment of tumor by taking advantage of the intracellular ingredients.

## Data Availability

The details regarding where data supporting reported results can be obtained from the authors.

## Supplementary Materials

The following are available online at https://www.mdpi.com/article/10.3390/nano11071843/s1, Figure S1: (A) SEM image of Cu-MOF. Scar bar: 50 nm. (B) Elemental mapping of C, N, O and Cu of Cu-MOF, Figure S2. (A) FT-IR spectra of Cu-MOF, Cu-MOF/GOD, Cu-MOF/GOD@HA and GOD. (B) TGA curves of Cu-MOF and Cu-MOF/GOD@HA, Figure S3. XRD spectra of Cu-MOF, Cu-MOF/GOD and Cu-MOF/GOD@HA and simulated XRD pattern of Cu-MOF, Figure S4. UV-vis absorption spectra of Cu-MOF and Cu(Ⅰ)-MOF (The concentrations of pending test samples were 100 μg mL^–1^). Inset image shows the photographs of the solutions containing Cu-MOF and Cu(Ⅰ)-MOF, Figure S5. TEM image of Cu-MOF after treatment by GSH, Figure S6. XPS pattern of Cu-MOF (A), Cu-MOF/GOD (B), Cu-MOF/GOD@HA (C), Cu(I)-MOF (D). Cu 2p high-resolution XPS pattern of Cu(I)-MOF (E), Cu-MOF (F), Cu-MOF/GOD (G), Cu-MOF/GOD@HA (H). Figure S7. UV-vis spectra of Cu-MOF+H_2_O_2_+MB and MB. Figure S8. (A) GSH depletion after incubation for 1 h in the presence of DTNB (720 μg mL^–1^) and Cu-MOF (0, 5, 15, 25, 50 μg mL^–^^1^). (B) The time-dependent reaction of Ampliflu Red solution (600 μg mL^–^^1^) with Glu (500 μg mL^−1^)+Cu-MOF/GOD (100 μg mL^–1^). (C) The concentration-dependent reaction of Ampliflu Red solution (600 μg mL^–1^) with Glu+Cu-MOF/GOD (100 μg mL^–1^) for 30 min (λ_ex_/λ_em_ = 530/585 nm). Figure S9. (A) The pH values of Cu-MOF+Glu and Cu-MOF/GOD+Glu aqueous solution in different time intervals. Cu-MOF and Cu-MOF/GOD: 100 μg mL^–1^, Glu: 500 μg mL^–1^. (B) The pH values of Cu-MOF and Cu-MOF/GOD with different concentration of Glu aqueous solution after 24 h. Cu-MOF/GOD: 100 μg mL^–1^, Glu: 0, 20, 50, 100, 500, 750, 1000 μg mL^–1^. Figure S10. ROS staining in MCF-7 cells after incubation with Cu-MOF, Cu-MOF/GOD and Cu-MOF/GOD@HA for 4 h at the concentration of 50 µg mL^–1^, scale bar: 10 µm. Figure S11. In vivo CDT treatment for MCF-7 cancer cell-bearing mice with different nanocompesites (PBS, Cu-MOF, Cu-MOF/GOD and Cu-MOF/GOD@HA 2.5 mg kg^–1^). (A) The changes of body weight for the KunMing mice during the process of therapy. (B) The variation of tumor size for the KunMing mice during the process of therapy. (C) The average relative mass excised from MCF-7 tumor-bearing mice after the treatment. (D) Photographs showing the tumor size after the treatment. Scale bar, 1 cm. (E) H&E staining of the major organs/tissues of mice after CDT process. Scale bar, 50 µm. Values of *p* < 0.05 were considered statistically significant, with ∗, ∗∗, ∗∗∗ represent *p* < 0.05, *p* < 0.01, and *p* < 0.001, respectively. 1, 2, 3 and 4 represent the group of PBS, Cu-MOF, Cu-MOF/GOD and Cu-MOF/GOD@HA, respectively. Figure S12. Hemolysis percentage of RBCs by Cu-MOF/GOD@HA nanodots at various concentration levels (10–100 µg mL^–1^). Inset: the photographs for direct observation of hemolysis.
