# Supplementary material for: Construction of Novel Nanocomposites (Cu-MOF/GOD@HA) for Chemodynamic Therapy
Source: Nanomaterials (Basel). 2021 Jul 16;11(7):1843. doi: 10.3390/nano11071843 (PMC8308241; doi:10.3390/nano11071843)
Supplement: Supplementary file 1 [file nanomaterials-11-01843-s001.zip › nanomaterials-1285701-supplementary.pdf]

Supplementary Materials

Construction of Novel Nanocomposites (Cu-MOF/GOD@HA) for Chemodynamic Therapy

Ya-Nan Hao [1,†], Cong-Cong Qu [1,†], Yang Shu [1,*], Jian-Hua Wang [1,*] and Wei Chen [2,3,*]

1 Department of Chemistry, College of Sciences, Northeastern University, Shenyang 110819, China; neuhaoyanan@163.com (Y.-N.H.); qcc1531486377@163.com (C.-C.Q.)
2 Departments of Physics, University of Texas at Arlington, Arlington, TX 76019, USA
3 Medical Technology Research Centre, Chelmsford Campus, Anglia Ruskin University,
Chelmsford CM1 1SQ, UK
* Correspondence: shuyang@mail.neu.edu.cn (Y.S.); jianhuajrz@mail.neu.edu.cn (J.-H.W.); weichen@uta.edu (W.C.)
† These authors contributed equally to this work

Chemicals and reagents.

3-(4, 5-dimethylthiazol-2-yl)-2, 5-diphenyltetrazolium bromide (MTT) was purchased from KeyGEN BioTECH Co., Ltd. (Nanjing, China). Methylene blue was obtained from Beijing Chemical Works Co., Ltd. (Beijing, China). Dulbecco's modified Eagle's medium (DMEM) and fetal bovine serum (FBS) were received from MeiLun biotechnology Co., Ltd. (Shenyang, China). Reduced GSH assay kit and ROS assay kit were acquired from Beyotime Co., Ltd. (Shanghai, China). Penicillin/streptomycin were purchased from HyClone Thermo Scientific (Logan, Utah, USA).H_2O_2 and 5,5-Dithio bis-(2-nitrobenzoic acid) (DTNB) was purchased from Aladdin Reagent (Shanghai, China). Copper(Ⅱ) acetate and benzoic acid was purchased from Sinopharm Chemical Reagent Co., Ltd. (Shanghai, China). 1,3,5-benzenetricarboxylic acid was purchased from Mayan reagent. (Zhejiang, China). Glucose oxidase was

purchased from Yuanye Biological Technology Co., Ltd. (Shanghai, China). Oligohyaluronic acid was purchased from Bloomage Freda Biopharm Co., Ltd. (Jinan, China). Glucose (Glu) was purchased from Damao Chemical Reagent Factory. (Tianjin, China). Ampliflu Red was purchased from Macklin Biochemical Co., Ltd. (Shanghai, China).

Instrumentation.

A FEI Tecnai G2 F20 transmission electron microscope (Philips, Holland) was used for TEM imagines of Cu-GA. X-ray diffraction (XRD) patterns were obtained on a D8 Advance diffractometer by using Cu Kα radiation at λ 1.54 Å (Bruker, Germany). Zeta potential and mean hydrodynamics and diameter values of the nanoparticles were measured by the Nano ZS/ZEN3690 instrument (Malvern, England). UV-vis absorption spectra were recorded on a U-3900 spectrophotometer (Hitachi High Technologies, Japan). Thermo gravimetric analysis of the nanospheres is performed on a 290C analyzer (TGA, Netzsch Company, Germany). FT-IR spectra is acquired on a Nicolet 6700 spectrophotometer (Thermo Electron, USA). X-ray photoelectron spectroscopy (XPS) analyses are carried out on an ESCALAB 250 Xray photoelectron spectrometer (Thermo Ltd., U.S.A.).

Mouse Tumor Model and Treatment.

Nude mice (6 weeks) provided by Hua Fu Kang Company (Beijing, China) were used for testing the anti-tumor effect of Cu-MOF/GOD@HA *in vivo*. The

procedures for animal experiments were conducted by strictly following "Animal and Medical Ethics Committee of Northeastern University" and the national standards "Laboratory Animal Requirements of Environment and Housing Facilities (GB14925-2001). For anti-tumor administration *in vivo*, 2×10^6 MCF-7 cells mixed with matrix glue (100 μL, 1:1) were subcutaneously injected into the right flank of each mouse. When the tumor volume reached ~90 mm^3, the mice were randomly divided into 3 groups (n=3) for anti-tumoral studies. The tumor bearing mice were treated with (1) PBS saline, (2) Cu-MOF, (3) Cu-MOF/GOD, (4) Cu-MOF/GOD@HA via intertumoral administration. These operations were repeated every 2 days by recording the body weight of the mice and measuring the tumor volume, which was quantified by the equation $V=0.5\ L\times S^2$, with L and S as the longest and the shortest dimension, respectively. The mice were sacrificed, the tumors and main organs were excised for further characterization on the 15th day after the first administration. The pictures and weight of the tumors were obtained before being fixed for H&E staining. For biosafety assessment, the major organs including heart, liver, spleen, lung and kidney, were sliced and analyzed using H&E staining.

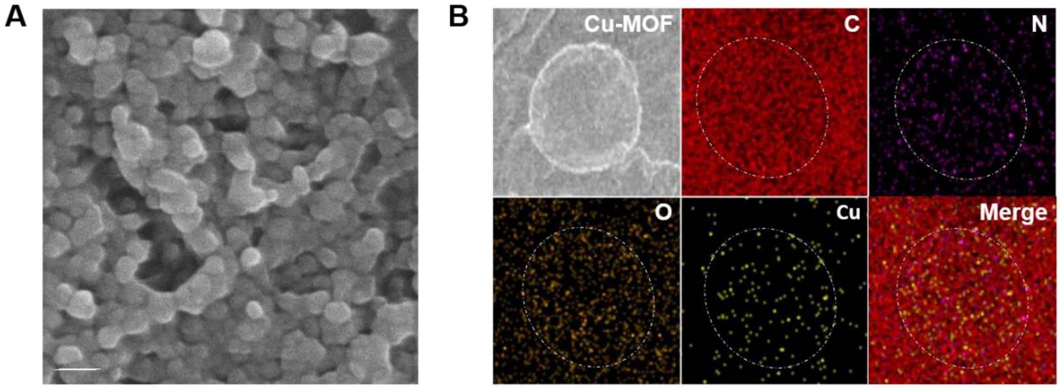

Figure S1. (A) SEM image of Cu-MOF. Scar bar: 100 nm. (B) Elemental mapping of C, N, O and Cu of Cu-MOF.

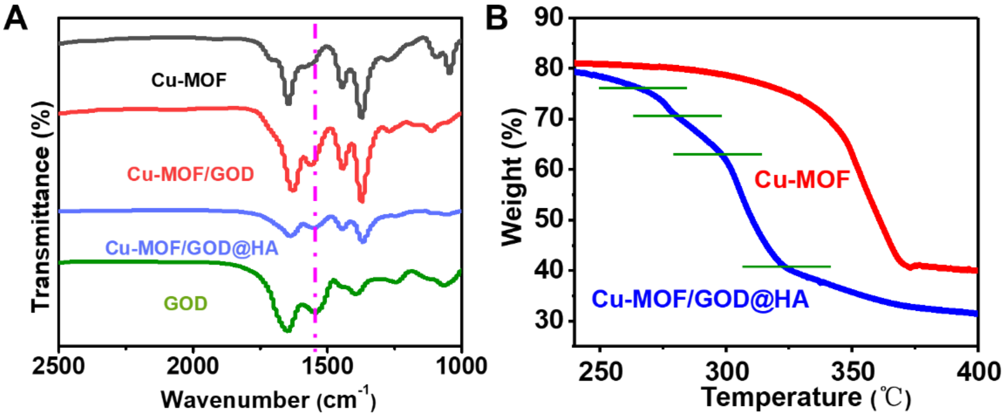

Figure S2. (A) FT-IR spectra of Cu-MOF, Cu-MOF/GOD, Cu-MOF/GOD@HA and GOD. (B) TGA curves of Cu-MOF and Cu-MOF/GOD@HA.

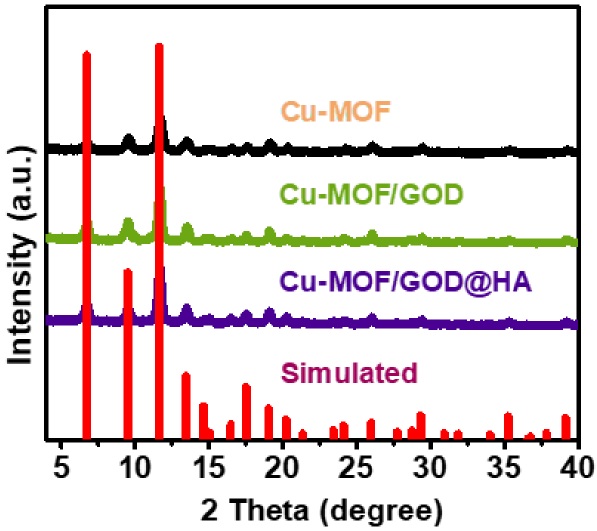

Figure S3. XRD spectra of Cu-MOF, Cu-MOF/GOD and Cu-MOF/GOD@HA and simulated XRD pattern of Cu-MOF.

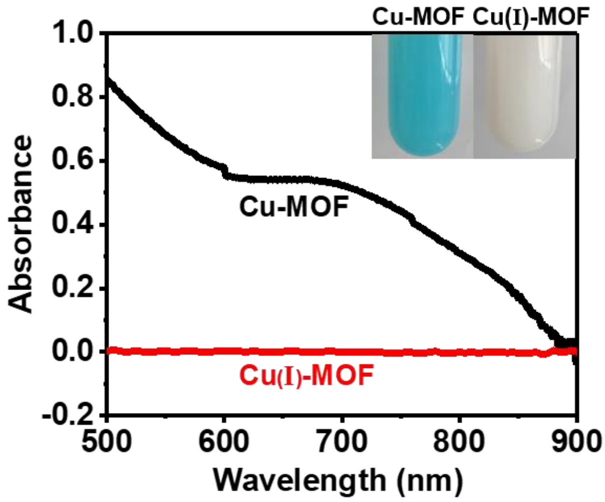

Figure S4. UV-vis absorption spectra of Cu-MOF and Cu(Ⅰ)-MOF (The concentrations of pending test samples were 100 μg mL^{-1}). Inset image shows the photographs of the solutions containing Cu-MOF and Cu(Ⅰ)-MOF.

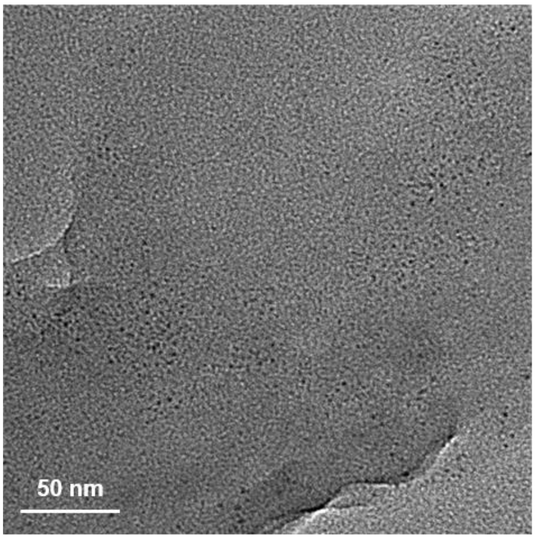

Figure S5. TEM image of Cu-MOF after treatment by GSH.

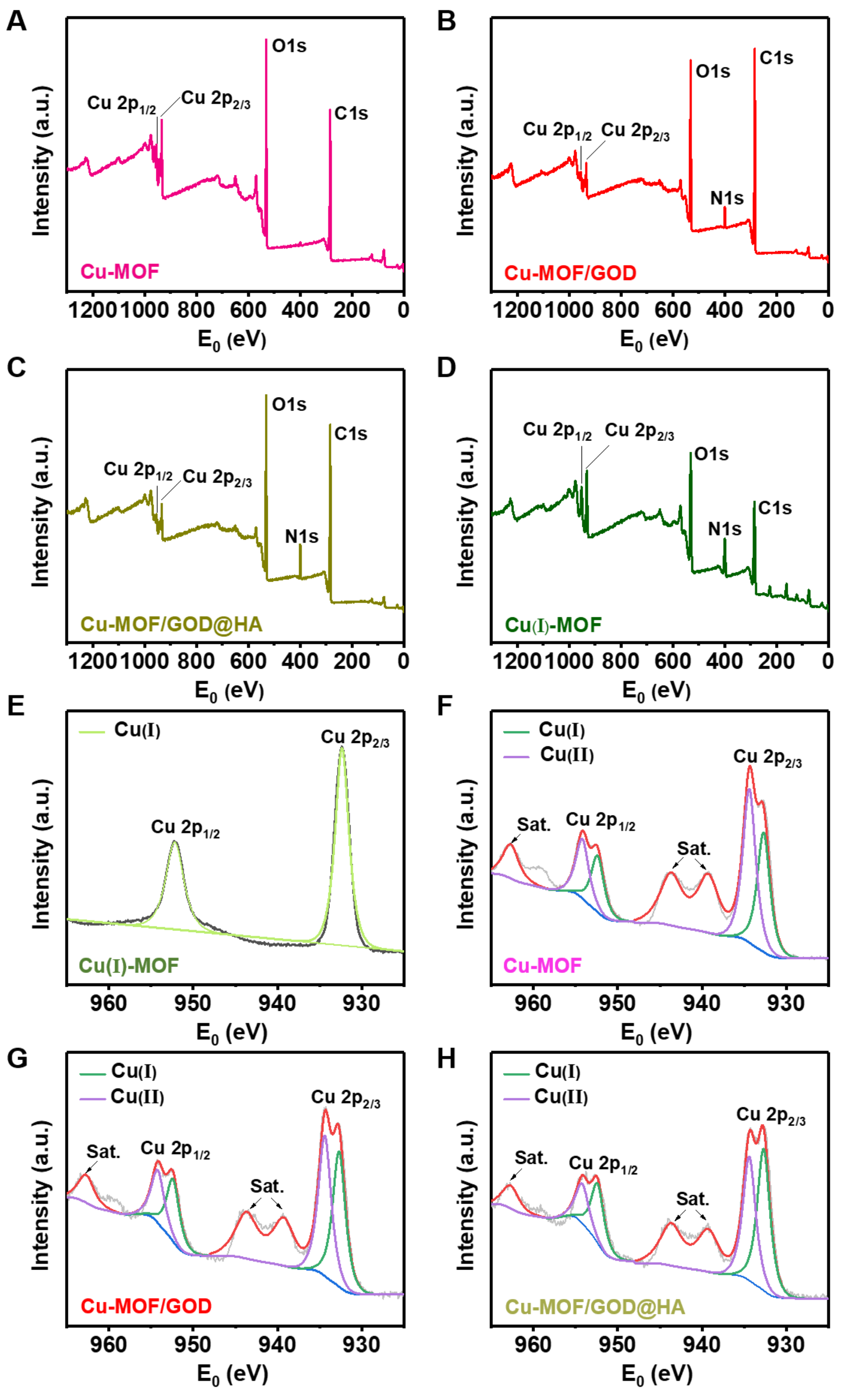

Figure S6. XPS pattern of Cu-MOF (A), Cu-MOF/GOD (B), Cu-MOF/GOD@HA (C), Cu(I)-MOF (D). Cu 2p high-resolution XPS pattern of Cu(I)-MOF (E), Cu-MOF (F), Cu-MOF/GOD (G), Cu-MOF/GOD@HA (H).

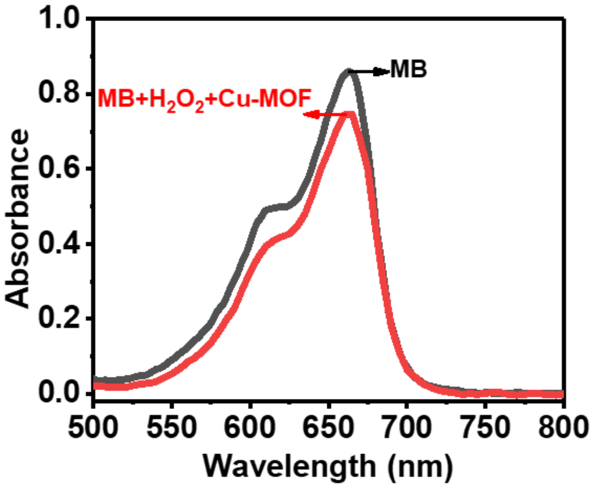

Figure S7. UV-vis spectra of Cu-MOF+H_2O_2+MB and MB.

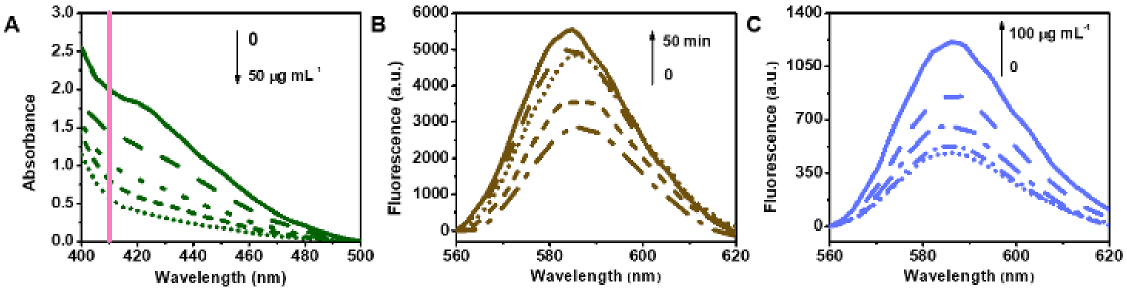

Figure S8. (A) GSH depletion after incubation for 1 h in the presence of DTNB (720 μg mL^{-1}) and Cu-MOF (0, 5, 15, 25, 50 μg mL^{-1}). (B) The time-dependent reaction of Ampliflu Red solution (600 μg mL^{-1}) with Glu (500 μg mL^{-1})+Cu-MOF/GOD (100 μg mL^{-1}). (C) The concentration-dependent reaction of Ampliflu Red solution (600 μg mL^{-1}) with Glu+Cu-MOF/GOD (100 μg mL^{-1}) for 30 min ($\lambda_{ex}/\lambda_{em}$ = 530/585 nm).

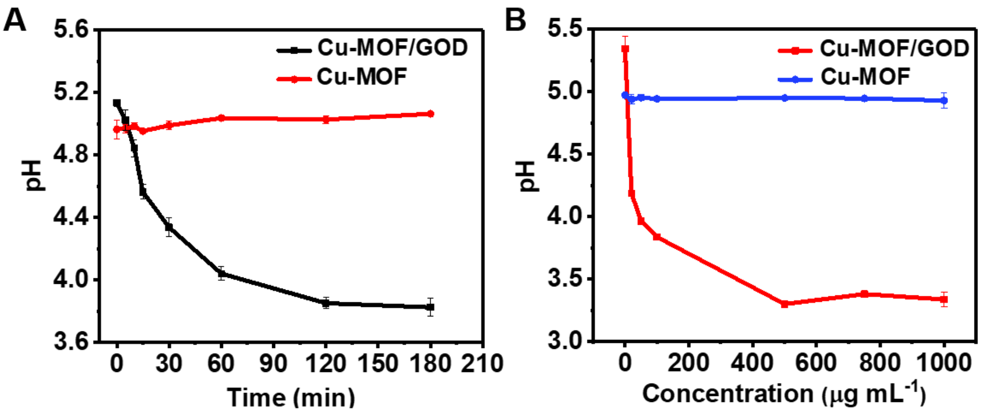

Figure S9. (A) The pH values of Cu-MOF+Glu and Cu-MOF/GOD+Glu aqueous solution in different time intervals. Cu-MOF and Cu-MOF/GOD: 100 μg mL^{-1}, Glu: 500 μg mL^{-1}. (B) The pH values of Cu-MOF and Cu-MOF/GOD with different concentration of Glu aqueous solution after 24 h. Cu-MOF/GOD: 100 μg mL^{-1}, Glu: 0, 20, 50, 100, 500, 750, 1000 μg mL^{-1}.

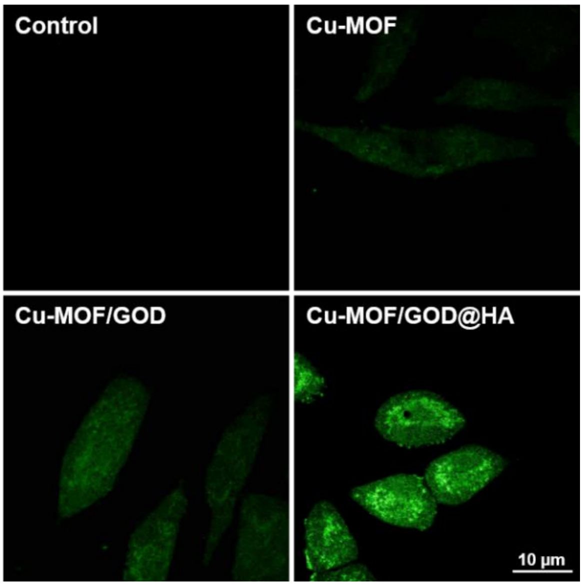

Figure S10. ROS staining in MCF-7 cells after incubation with Cu-MOF, Cu-MOF/GOD and Cu-MOF/GOD@HA for 4 h at the concentration of 50 μg mL^{-1}, scale bar: 10 μm.

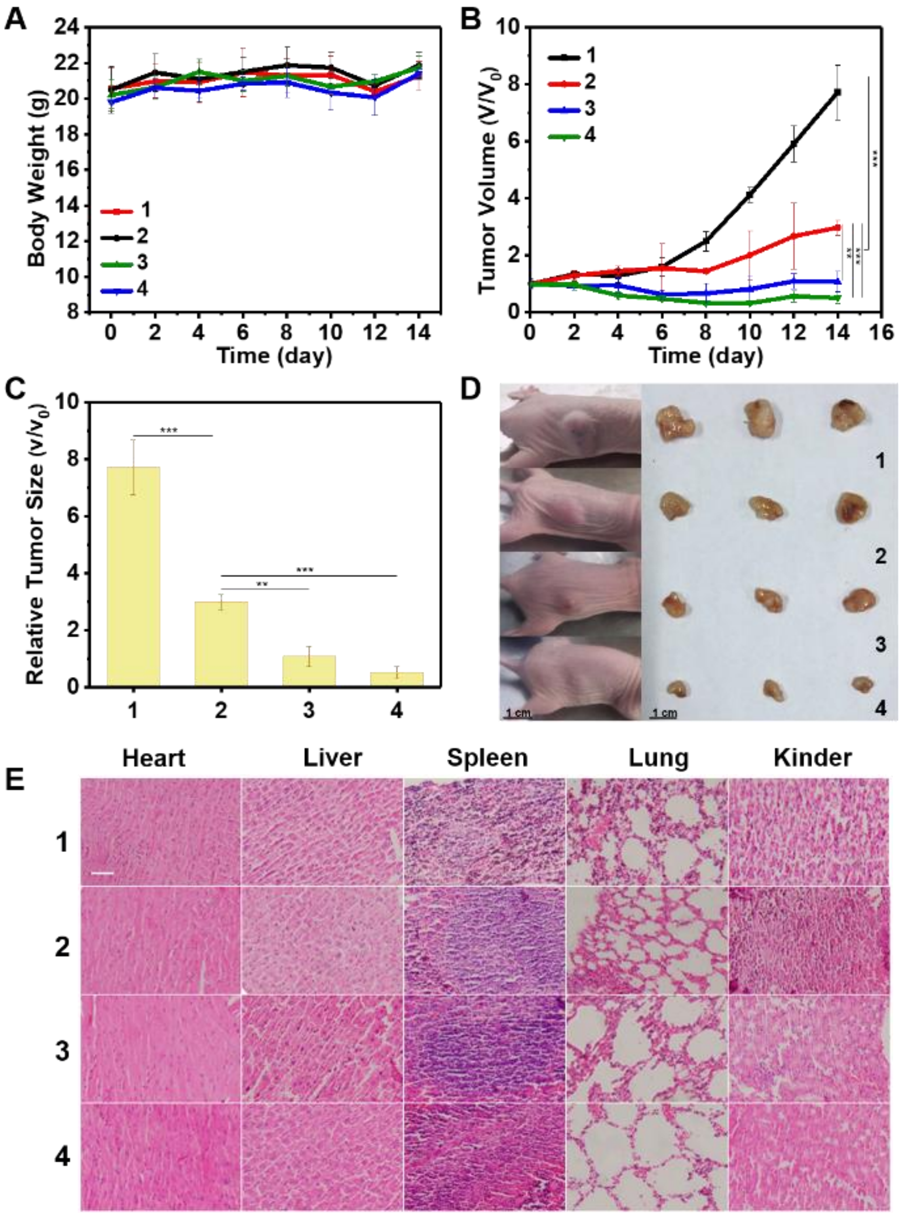

Figure S11. In vivo CDT treatment for MCF-7 cancer cell-bearing mice with different nanocomposites (PBS, Cu-MOF, Cu-MOF/GOD and Cu-MOF/GOD@HA 2.5 mg kg^{-1}). (A) The changes of body weight for the KunMing mice during the process of therapy. (B) The variation of tumor size for the KunMing mice during the process of therapy. (C) The average relative mass excised from MCF-7 tumor-bearing mice after the treatment. (D) Photographs showing the tumor size after the treatment. Scale bar, 1 cm. (E) H&E staining of the major organs/tissues of mice after CDT process. Scale bar, 50 μm. Values

of $P<0.05$ were considered statistically significant, with *, **, *** represent $p<0.05$, $P<0.01$, and $P<0.001$, respectively. 1, 2, 3 and 4 represent the group of PBS, Cu-MOF, Cu-MOF/GOD and Cu-MOF/GOD@HA, respectively.

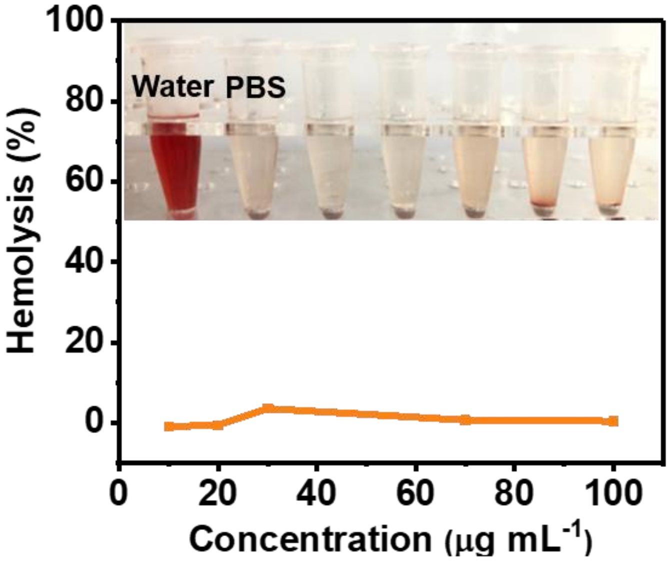

Figure S12. Hemolysis percentage of RBCs by Cu-MOF/GOD@HA nanodots at various concentration levels (10-100 μg mL^{-1}). Inset: the photographs for direct observation of hemolysis.
